# KAUST Metagenomic Analysis Platform (KMAP), enabling access to massive analytics of re-annotated metagenomic data

**DOI:** 10.1038/s41598-021-90799-y

**Published:** 2021-06-01

**Authors:** Intikhab Alam, Allan Anthony Kamau, David Kamanda Ngugi, Takashi Gojobori, Carlos M. Duarte, Vladimir B. Bajic

**Affiliations:** 1 Computational Bioscience Research Center, King Abdullah University of Science and Technology (KAUST), Thuwal, 23955-6900 Saudi Arabia; 2 Leibniz Institute DSMZ-German Collection of Microorganisms and Cell Cultures, Inhoffenstraße 7B, 38124 Brunswick, Germany; 3 Red Sea Bioscience Research Center, King Abdullah University of Science and Technology (KAUST), Thuwal, 23955-6900 Saudi Arabia

**Keywords:** Computational platforms and environments, Metagenomics, Data integration

## Abstract

Exponential rise of metagenomics sequencing is delivering massive functional environmental genomics data. However, this also generates a procedural bottleneck for on-going re-analysis as reference databases grow and methods improve, and analyses need be updated for consistency, which require access to increasingly demanding bioinformatic and computational resources. Here, we present the KAUST Metagenomic Analysis Platform (KMAP), a new integrated open web-based tool for the comprehensive exploration of shotgun metagenomic data. We illustrate the capacities KMAP provides through the re-assembly of ~ 27,000 public metagenomic samples captured in ~ 450 studies sampled across ~ 77 diverse habitats. A small subset of these metagenomic assemblies is used in this pilot study grouped into 36 new habitat-specific gene catalogs, all based on full-length (complete) genes. Extensive taxonomic and gene annotations are stored in Gene Information Tables (GITs), a simple tractable data integration format useful for analysis through command line or for database management. KMAP pilot study provides the exploration and comparison of microbial GITs across different habitats with over 275 million genes. KMAP access to data and analyses is available at https://www.cbrc.kaust.edu.sa/aamg/kmap.start.

## Introduction

The diversity of microbes has been extrapolated to an estimate in the order of 1 trillion species worldwide^1^. To understand their roles, two key questions must be answered: ‘Who are they?’ and ‘What are they doing?’. Metagenomics is a suitable approach to provide these insights^2,3^, leading to concerted efforts in order to cope with the scale and complexity of the problem. The Earth Microbiome Project addresses ‘Who are they?’ by performing a massive taxonomic analysis on environmental samples^4^. Hence, addressing the question ‘What are they doing?’ remains an outstanding challenge. Addressing this question requires an understanding of the functional roles of microbes, through the capabilities encoded in their genetic material^5^, and its potential applications in research and industry. Microbial metagenomics, involving massive shotgun sequencing of microbial communities, was introduced in 1998^6^, as a powerful approach to address both questions simultaneously^7^. Since then the volume of shotgun metagenomic samples available for analyses has increased exponentially, propelled by the sharp decline in sequencing costs^8^, amounting to Petabyte scale data of DNA, and RNA, sequences^9^. These data, along with extensive metadata for samples, are available through the European Nucleotide Archive (ENA), where the European Bioinformatics Institute (EBI) metagenomics server^10^ provides annotation capabilities.

To compare and make use of older and recent shotgun metagenomic studies alike, given advancements in metagenomic assembly methods^11–13^ and continued improvements in reference databases, on-going re-assembly and re-analysis of samples using state-of-the-art methods is needed. Previously, gene prediction and analysis were derived directly from short reads, resulting in a large collection of broken genes. In recent studies, assembly of the metagenomes is performed before further analysis; however, there are no filters applied for using only the complete genes. We propose metagenomic gene prediction on assembled data to include only complete genes. It helps in many ways e.g. when sequencing technology changes for longer reads, where assembly of the data may not be required, existing analysis from assembly based complete genes will not be lost. Moreover, partial genes make functional validation of interesting genes nearly impossible, therefore an analysis based on full length genes is more tractable.

Metagenomic assembly-based analysis can lead to the recovery of more complete genes, better annotation coverage, and insights into the microbial world, however, it is computationally very expensive. Here, a comparative metagenomics approach using clustering of full-length genes from multiple samples, producing a gene catalog matrix of genes vs samples, can help reduce the computational load of the annotation process and the redundancy of genes present in multiple related samples. In this approach gene-abundance estimates from reads are mapped onto a common gene catalog instead of genes from each sample, similar to gene catalogs developed by the Human Integrated Gut (HIG) Microbiome study^14^ and *Tara* Oceans^15^. An additional benefit of full-length genes obtained from gene catalogs, compiled using metagenomes assembled from diverse environments, is an opportunity to functionally catalogue genes present across the biosphere. Such a gene pool, coupled with sample metadata (e.g., temperature and salinity), can serve as a basis to accelerate discovery and applications for industries such as biotechnology, pharmaceutics, food and energy, and others. This re-analysis is, however, challenging as it requires advanced bioinformatics skills and computational resources in order to process all existing and new metagenomic samples through state-of-the-art methods to assemble and predict genes and metabolic processes, clustering and functional annotation with updated reference databases. Another major challenge is the lack of standards in metagenomic data analysis, reporting and data sharing for reproducibility, repurposing and reuse^16^.

Here we describe the KAUST Metagenomic Analysis Platform (KMAP) allowing annotation and comparison of massive metagenomic datasets included with a few examples, screencast videos and associated methods with a concept of improved metagenomic analyses.

## Results and analyses

**Figure 1:**
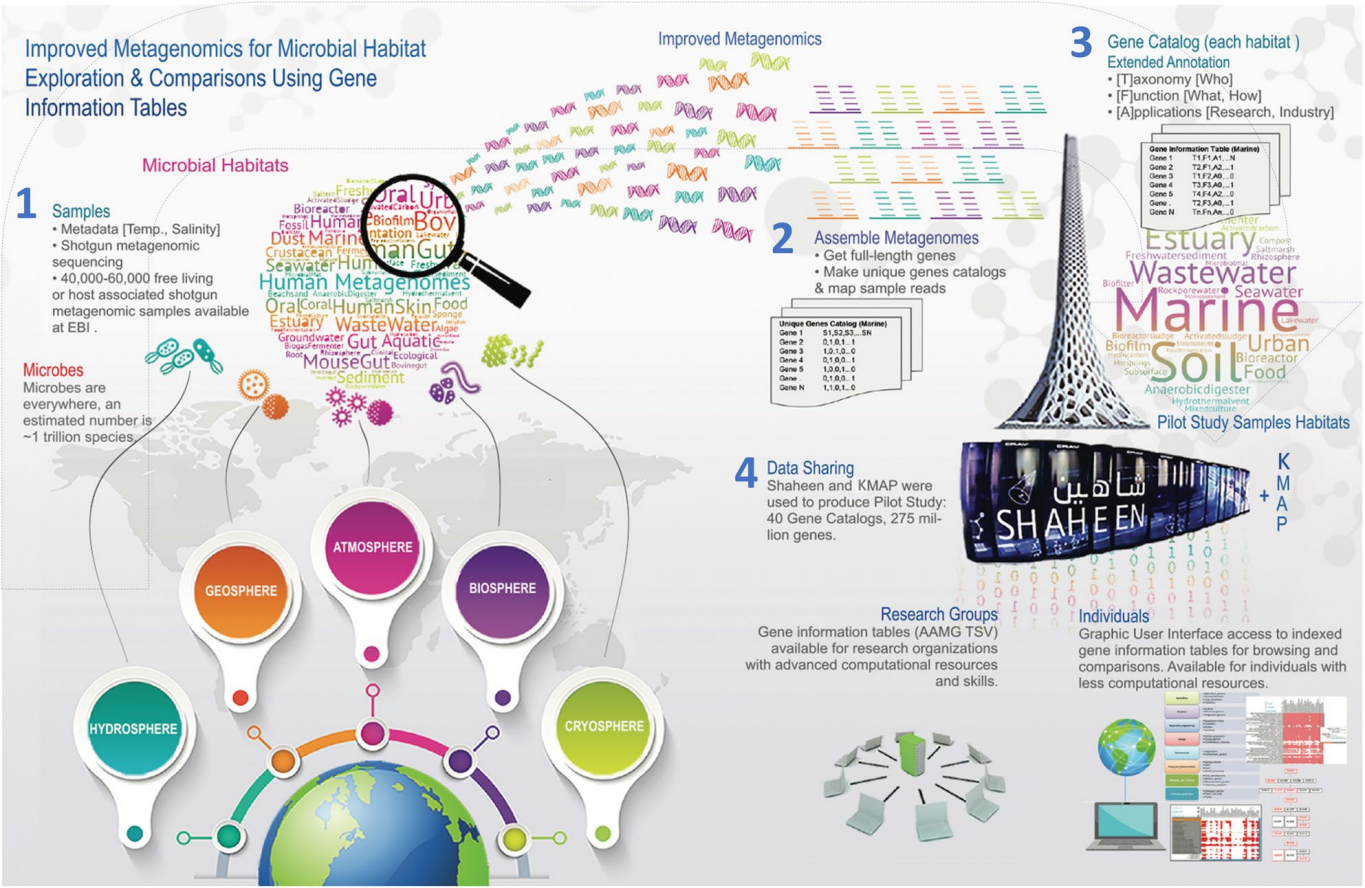
A summary of the KAUST Metagenomic Analysis Platform (KMAP) concept is as follows: (1) utilize existing short reads-based samples from European Nucleotide Archive (ENA), (2) improve shotgun metagenomic analysis through assembly and organization of full-length (complete) genes into habitat-specific gene catalogs (see pilot studies), and (3) extending and improving annotations of gene catalogs using KMAP providing Gene Information Tables (GITs) useful for data sharing with research groups for further computing intensive command-line analyses or individuals with fewer computational resources. Example gene catalogs reported in this study are shown in Supplementary Table ST1.

To perform metagenomic annotation and comparisons through KMAP (section e), we first describe the concept of improved metagenomics, as shown in Fig. 1. In this concept we propose (re)assembly of existing short reads based metagenomic samples and prediction of only full-length (complete) genes leading to non-redundant gene catalogs. These gene catalogs are then annotated by KMAP annotation module using recent reference databases. This work includes a pilot study on annotation of 40 gene catalogs. These are made publicly available for interactive online comparisons as well as for download. In the sections below, we report as a requirement for improved metagenomics (a) the re-assembly of public metagenomic samples; (b) creation of gene catalogs from diverse environments using subset of assemblies, as a pilot study; (c) re-annotation of existing gene catalogs for improved coverage; (d) the design of Gene Information Tables (GITs) to standardize shotgun metagenomic analysis, reporting and allowing reuse; followed by (e) KAMP annotation and exploration methods compared to other existing platforms allowing metagenomics analysis; and (f) KAMP capacities for targeted comparison and interrogation of key genes of interest such as antibiotic resistance genes (ARGs) from different environments accessible through microbial gene catalogues in KMAP database.

### Re-assembly of public metagenomic samples

A large number of metagenomic samples based on short reads sequencing were deposited to public repositories over the last decade. Their associated analyses in most cases were based on either directly using short reads for gene predictions or most recently employing the assembly^10^ step but including a mix of both partial and complete genes. To obtain full-length (complete) genes from existing studies it is worthwhile to harmonize gene reconstruction protocols, particularly the computationally demanding metagenomic assembly procedure equally to all samples. To compile a list of publicly available shotgun metagenomic datasets we searched ENA database (accessed May 2018) and retrieved ~ 27,000 samples (out of a list of over 34,000) with a valid ftp download location. The shotgun microbial metagenomic samples were grouped according to their designated environments or habitats as available in ENA (complete details are provided in Supplementary Tables ST1, ST2). The metagenomes were processed through a quality control protocol and validation of read pairs, followed by the assembly of individual samples in paired-end mode (see “Materials and methods” section). For easy access to sample metadata, we include references of ENA identifiers representing sample taxon id, taxon name, study or project identifier and run accession identifiers in the header of assembly sequence files and assembly statistics as html tables (see download section of KMAP website). Crucially, these datasets are available to the wider scientific community for further targeted analyses precluding the need to retrieve and re-assemble the original reads. As a pilot study, a subset of these assemblies are compiled into series of gene catalogs from diverse environments extensively annotated with taxonomic and gene function, as described below.

### Creating habitat-specific gene catalogs

A large set of metagenome assemblies were produced in this work (see Supplementary Materials), however our focus here is to use, as a pilot study, a subset of assemblies to produce full length (complete) genes based gene catalogs from a few ecological environments based on harmonized procedures. For this purpose we processed a set of samples from each of the main biomes using Prodigal gene prediction method^17^ with an option “-c” that allows predicting genes with closed ends, avoiding gene prediction near the end of contigs. We generated gene catalogs from a few ecological metagenomes encompassing 36 out of 77 environments reported in ENA projects (called studies). Genes obtained from all samples covering the same habitat category were combined into a single set, followed by a clustering procedure to create each of the 36 habitat-specific gene catalogs. For clustering, we used CD-HIT^18^ software (https://github.com/weizhongli/cdhit/releases/tag/V4.6.8), with two global nucleotide sequence identity cutoffs of 95% and 90% of the query gene sequence. For both identity cutoffs, the alignment coverage was capped at 80% of the query gene length to avoid clustering shorter genes with longer ones. Supplementary Table ST1 shows a list of microbial habitats, number of samples included, ENA study ids, number of unique genes, and pertinent annotation information that we explain below.

#### A global non-redundant microbial metagenomic reference gene catalog

A global microbial metagenomic reference gene catalog (KMAP global meta-proteome) was produced from all habitat-specific non-redundant gene catalogs at the protein level containing 275 million genes. For this purpose, we used the MMseq2^19^ clustering approach, applying a percent global sequence identity of 90% and minimum gene length difference of 80%. The resultant global non-redundant microbial gene catalog is composed of 177.4 million proteins; see Supplementary Fig. S1 that shows the percentage of unique and common peptide sequences across different gene catalogs used in producing this gene catalog. The global microbial reference gene catalog contains non-redundant proteins across diverse habitats available as reference dataset for direct annotation of new metagenomic samples. It is available online, alongside other gene catalogs reported in this study, for sequence comparison through BLAST, at KMAP website, as well as a FASTA formatted sequences data file, available at http://www.cbrc.kaust.edu.sa/aamg/KMAPglobalRef/KMAP_Global_MetaProteome__proteins_NR.fasta.gz.

### Annotation of gene catalogs with improved coverage

**Figure 2:**
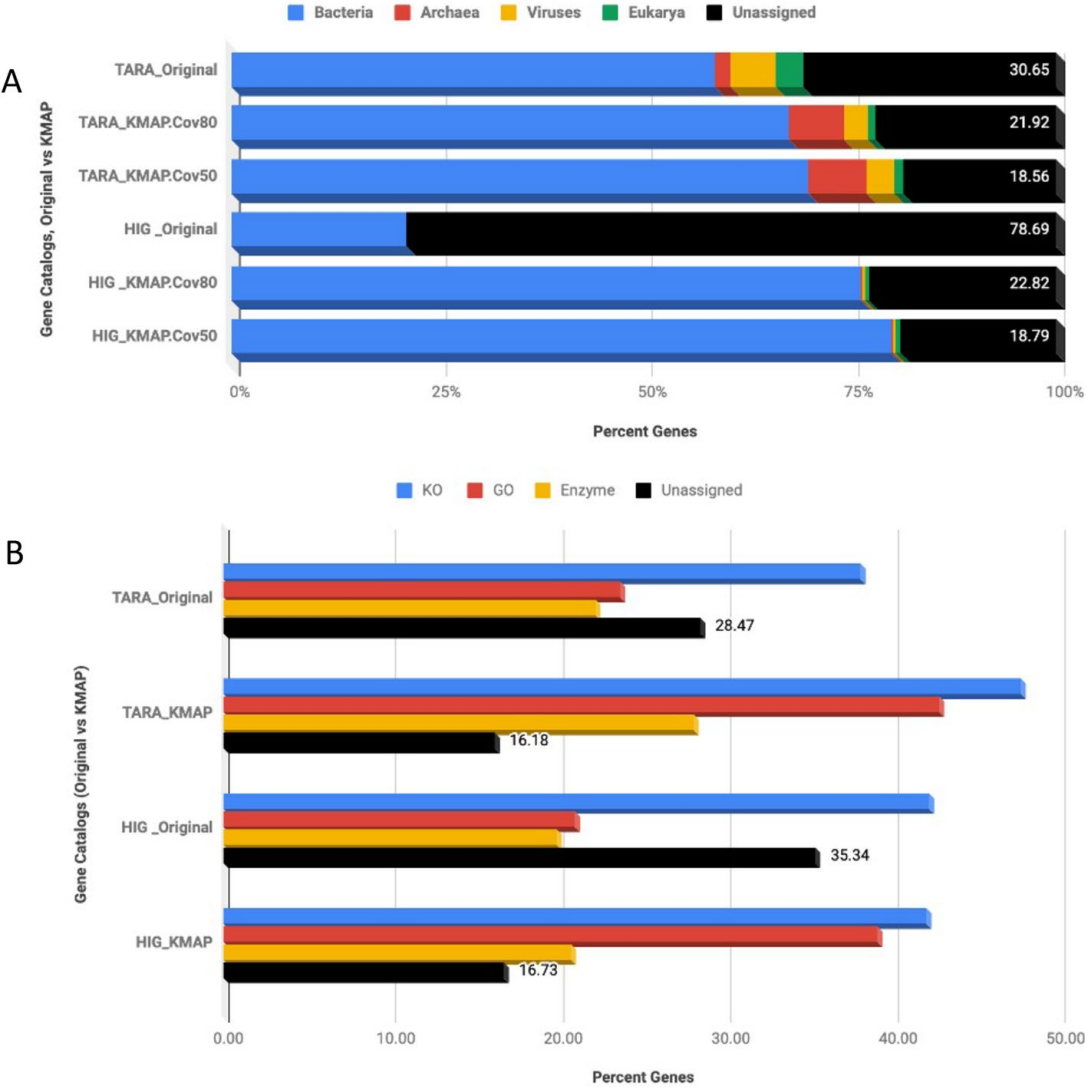
Examples of improved results in gene assignment. (**A**) Taxonomic coverage of existing gene catalogs, original versus re-annotation. (**B**) Functional coverage of public gene catalogs, original (2012) versus re-annotation (2018). *HIG* human integrated gut; Cov80 and Cov50 mean coverages of 80% and 50%, respectively.

Given the continued update and improvement of relational public reference databases critical for gene annotation, we posit that previously annotated metagenomes or gene catalogs can be significantly improved and anchored with up-to-date taxonomic and functional annotation. For instance, the massive gene catalogs from the Human Integrated Gut (HIG)^14^ (~ 10 million genes) and the *Tara* Ocean’s marine metagenome^15^ (~ 40 million genes) sampling programs significantly improved when re-annotated using updated reference databases regarding the proportion of taxonomically and functionally assigned genes in comparison to results reported just a few years ago (Fig. 2). These two gene catalogs were previously annotated in the year 2012 when the total number of reference sequences in Universal Protein Knowledgebase was around 25 million; this number now increased to over 175 million. Similarly, there were ~ 16,000 KEGG Ortholog (KO) families available in KEGG database in 2012 that number now increased to over 22,000 (see https://www.genome.jp/kegg/docs/upd_all.html).

We performed extended annotation of genes for taxonomic assignment and functional annotation. Protein sequences were compared to the Universal Protein Knowledgebase (UniProKB www.uniprot.org) reference database to infer taxonomic origin of the gene sequences, and also cross references to e.g. Cluster of Orthologous Genes (COGS) and the eggNOG (eggnog.embl.de) datbase. Furthermore, genes were compared to the Kyoto Encyclopedia of Genes and Genomes (KEGG) database (www.kegg.jp) to infer KEGG Orthologies (KOs) of functional roles and enzyme information. Also, the InterPro database is used to obtain functional signature domains and Gene Ontology (GOs).

**Figure 3:**
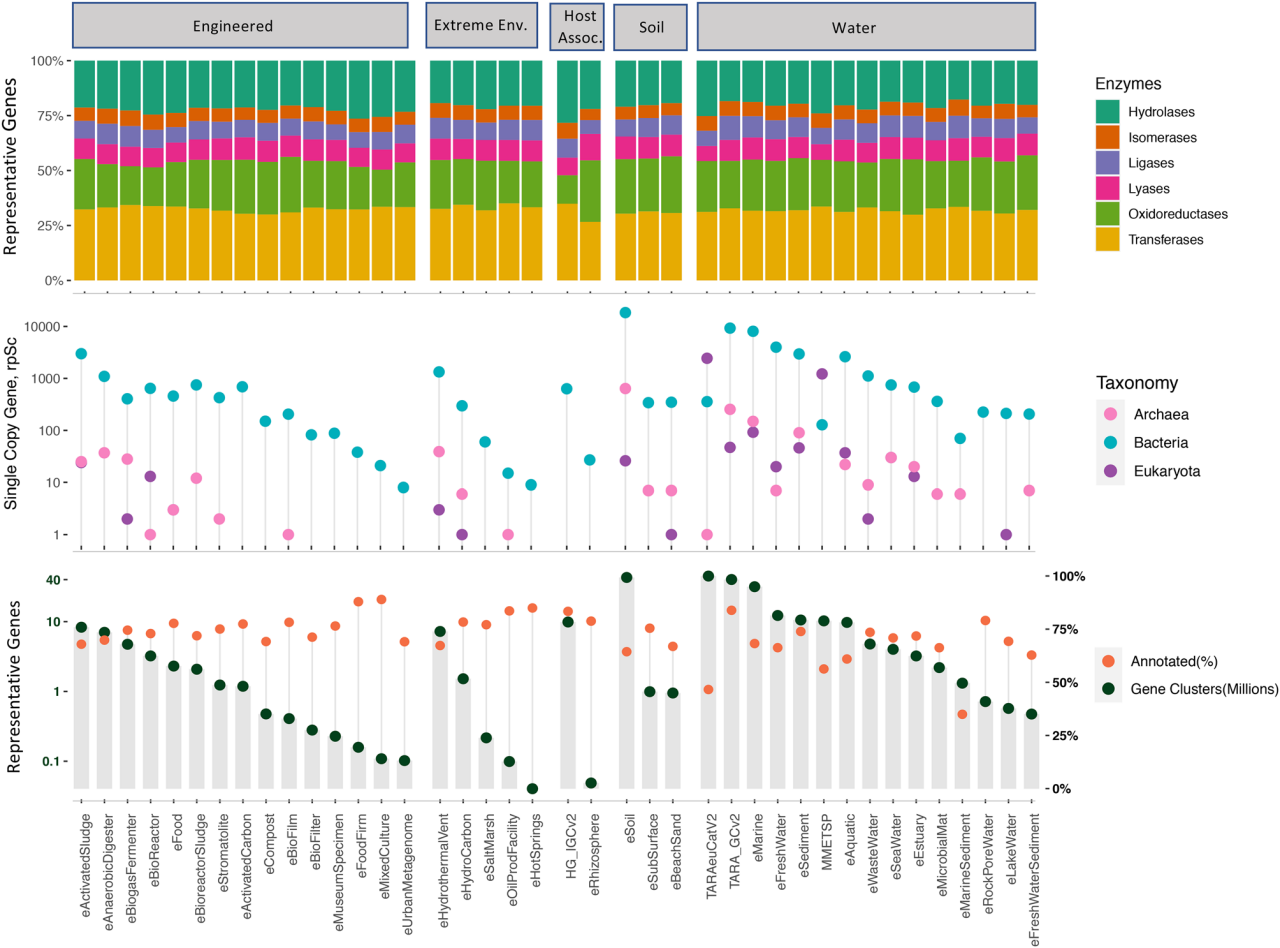
Gene catalogs showing count of habitat specific representative genes. (**A**) The count of total representative genes (million) as well as annotated genes (percent). (**B**) The count of taxon assigned single copy marker gene rpSc (PF01738) is shown on the log scale and (**C**) unique genes labeled with enzymes of different types. This data presents 36 new gene catalogs from ecological metagenomes, marked with initial “e”. Existing four gene catalogs are also included alongside the global metagenomic gene catalog, see Supplementary Table S1 for details on sample count, study accessions and corresponding annotation from KAMP.

In our analysis, massive improvement in the fraction of genes with assigned taxa was observed in the HIG gene catalogue under both stringent criteria versus default criteria (80% vs. 50% BLAST query coverage) for taxonomic assignment using our approach. For instance, 77–81% of the genes were annotated relative to the original study^14^, where ~ 21.3% genes were assigned a taxonomic label, see Fig. 2 and http://meta.genomics.cn/meta/home. In the case of the *Tara* Ocean’s gene catalog, the re-annotation improved taxonomic coverage by ~ 10% with stringent coverage and ~ 12% with default coverage parameters (Fig. 3). Minimum percent identity is kept at 30% for amino acid sequence alignments. Regarding functional coverage, re-annotation reduced unassigned genes from 28.5 to 16.2% for *TARA* and 35% to 16.7% in the case of HIG (Fig. 2).

A further analysis of older and new version of annotations shows significantly higher proportion of genes in the *Tara* Ocean and HGM catalogs were functionally and taxonomically anchored with new labels following re-annotation (Table 1). For instance, about 3.9 million more genes were functionally assigned in the *Tara* gene catalog relative to the original annotations, while 4.8 million more genes received a new taxonomic label with re-annotation of the HGM gene catalog (9.8 million genes; Table 1). This represents an improvement of roughly 10 and 50% percent, respectively. In parallel, around 2066 (in *Tara*) and 2401 (in HGM) KO entries were added with re-annotation, while 218 (in *Tara*) and 1261 (in HGM) previously annotated KOs changed labels. Of note, is that the previous (2012) and new (2019) KEGG databases have overall 16,000 and 22,000 KO entries respectively. Similarly, 166 (in *Tara*) and 301 (in HGM) taxa (at the order rank) were added, with roughly 130 (in *Tara*) and only one (in HGM) changing assignment with re-annotation (Table 1). Considering only the previous KOs labels (7934 and 7599 in *Tara* and HGM, respectively) and corresponding assigned gene copies in the original and the re-annotated gene catalogs (Table S1A), indicates that the diversity of KOs is much higher in the original annotations. However, the represented KO diversity is more evenly distributed with re-annotation, presumably because of reassignment of previous genes with KO labels (total of 10,000 KOs) based on the updated KEGG database and improvement in gene assignment criteria with length and identity cutoffs rather than just bitscore values.

**Table 1:** Alpha diversity measures comparing original (v1) and re-annotated (v2) *Tara* Ocean and human gut microbiome (HGM) gene catalogs.

Total genes	KEGG assignments		Taxonomic assignments	
Alpha Div metrics	Tara_GCv1	Tara_GCv2	Tara_GCv1	Tara_GCv2
Catalogued (nr) genes	40,154,822	40,154,704	40,154,822	40,154,704
Shannon	7.60	**7.70**	**3.74**	2.99
Simpson	0.9992	**0.9993**	**0.9455**	0.8791
InvSimpson	1242.1	**1357.6**	**18.3**	8.3
Richness	**7934**	7716	**594**	464
# v1 entry labels	7934	7934	594	594
# v1 labels not in v2	NA	**218**	NA	**130**
# all labels per v2	10,000	10,000	760	760
# v2 labels not in v1	2066	NA	166	NA
# v1 genes assigned	15,257,684	**17,586,877**	13,570,297	**18,316,039**
# v2 genes assigned	15,257,684	**19,140,981**	13,570,297	**18,491,362**

	HG_IGCv1	HG_IGCv2	HG_IGCv1	HG_IGCv2
Catalogued (nr) genes	9,879,896	9,879,620	9,879,896	9,879,620
Shannon	7.49	**7.51**	**2.07**	1.78
Simpson	0.9988	**0.9990**	**0.7912**	0.6715
InvSimpson	858.8	**976.6**	**4.8**	3.0
Richness	**7599**	6338	**85**	84
# v1 entry labels	7599	7599	85	85
# v1 labels not in v2	NA	**1261**	NA	**1**
# all labels per v2	10,000	10,000	386	386
# v2 labels not in v1	2401	NA	301	NA
# v1 genes assigned	4,154,983	**3,683,439**	1,610,393	**6,367,492**
# v2 genes assigned	4,154,983	**4,161,405**	1,610,393	**6,431,446**

Higher metrics are shown in bold.

These improvements prompted us to address the issue of a more comprehensive annotation of the earlier shotgun metagenomic data sets. The aggregated global microbial gene catalog reached over 275 million non-redundant gene sequences. We performed these extended annotations for all of the 36 new and four existing gene catalogs, see Fig. 3 and Supplementary Table ST1 showing the proportion of annotated vs unannotated genes where new gene catalogs appear with an initial “e” denoting source of sequence data is ENA.

From the gene catalogs reported here, 16 are from Aquatic environments, 15 are from Engineered environments, 3 are from Soils and another 2 from host-associated environments. Annotation of these gene catalogs reveal (Fig. 3A) that over 50% of the genes were assigned a putative function except marine sediment and Tara eukaryotic gene catalogs. The annotation of the global non-redundant gene catalog (KMAP global meta-proteome) showed that ~ 57% of genes (~ 101 million genes) had probable functions. An open question, therefore, is how much microbial diversity is captured in the functional assignment. To estimate potential taxonomic diversity in metagenomic environments as operational taxonomic unit (OTU), one of the universal single copy gene such as ribosomal protein S30 (rpSc) can be used as a proxy^20^. Figure 3B shows taxonomic diversity across different habitats through count of variants, or OTUs based on rpSc gene, as assigned with a taxonomic label (considering BLAST based percent identity of 30 and percent coverage of 80 and presence of rpSc domain PF01738). The diversity across different gene catalogs summarized for the global gene catalog showed 34,471 different bacteria, 2430 Eukaryota and 700 different Archaea in this dataset. Highest taxonomic diversity appears to be in the soil metagenomes, followed by aquatic environments. From the engineered environments, activated sludge and anaerobic digester gene catalog showed taxonomic diversity above 1000 OTUs for different microbes.

Figure 3C summarizes count of unique genes related to different enzyme classes throughout different habitat-specific gene catalogs. Also included is an interactive heatmap of complete and incomplete KEGG pathway modules, see https://bit.ly/2WvVtLX for full interactive map. Here, the functional repertoire of individual microbial habitats, compared to all others, can be interactively tracked on the level of a single or set of critical genes required to activate portions of pathways.

A BLAST-based sequence comparison of the 275 million genes to reference databases, such as the UniProtKB, requires ~ 522 years of computer processing time using a single central processing unit (CPU) computer, however the same task on KAUST’s Shaheen II supercomputer completed sequence comparisons in ~ 13 days using ~ 4.8 million computer CPU hours per day.

### Gene Information Tables (GITs)

Gene Information Table (GIT) represent a simple tab separated text-based table, similar to the one introduced by Metagenomics Reports (MetaRep) framework^21^, showing unique types of annotations for a list of genes available from a sample, a gene catalog, a genome or a metagenome (see an example GIT in Supplementary Fig. S1). It includes annotations such as gene name, Gene Ontology (GO), Enzyme Classification (EC), InterPro domains, Taxon ID, Annotation type filters, KEGG Orthology (KO) ID, weight or an expression value, COG and eggNOG IDs. There is a source column to report the source of annotation and to later filter genes based on BLAST statistics, E-value, Percent Identity, Percent Coverage data are recorded. Interesting “Filters” can be introduced to work with subset sets of genes for example the ones available with KO, Enzyme, COG or other interesting sources.

GITs available for a genome, metagenome or a gene catalog can be easily used for further analysis and comparison of data sets using commandline tools. As the size of data grows in metagenomic analysis, it would require significant computing power and advanced computational skills to sift through these data sets for deeper analyses. On the other hand these GITs can be indexed into a database, e.g. using MetaRep framework^21^, for easy web-based browsing, querying and comparisons of taxonomic or functional aspects of different metagenomic datasets. In this study we performed a large number of metagenomic assemblies from different environments; these data sets are openly available for public use through their choice of annotation and analyses with a recommendation to use GIT format for data sharing and data integration. Example GITs related to gene catalogs reported here are available (see Table ST1, for download or building a database).

### An example database to explore and compare GITs from shotgun metagenomic data

Considering the increasing volume of data, a normal user may not be able to process these huge shotgun metagenomics datasets for further analyses; however, using GITs data integration, further analyses become easy, either via commandline methods or through a database. Here, we present the KAUST Metagenomic Analysis Platform (KMAP) an open-access platform providing wider access for the annotation (producing GITs), exploration and comparison (by providing a database) of microbial shotgun metagenomic data.

### KAUST Metagenomic Analysis Platform (KMAP)

**Figure 4:**
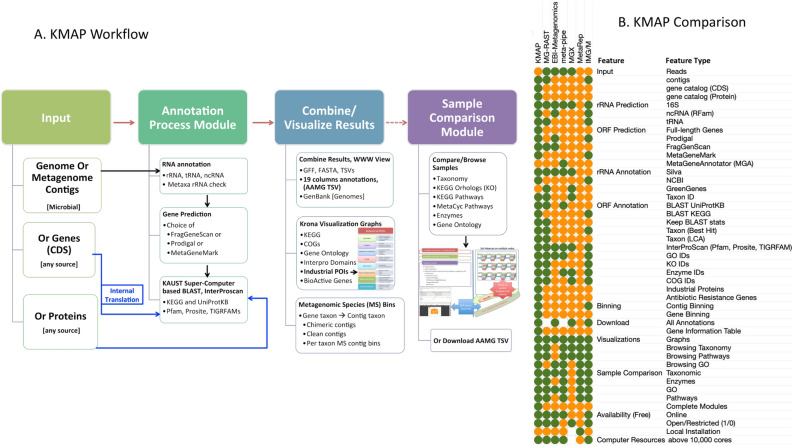
KMAP workflow and Comparative overview of features in KMAP and other most relevant pipelines for metagenomic data analysis. The pipelines included in comparison are MG-RAST^23^, EBI Metagenomics^10^, Meta-Pipe^24^, MGX^25^, MetaRep^21^ and IMG/M^26^. A score of 1 is assigned for features present (green color), 0 for absent (orange) and with limited availability a score of 0.5 is assigned (white).

KMAP consists of two modules: The Annotation Module, which is used for annotation of user-submitted contigs or genes, and the Compare Module, which allows for sample-to-sample or gene catalog-based comparison (see Fig. 4A, “Materials and methods” section and KMAP documentation). The annotation process and compilation of GITs is implemented in the Automatic Annotation of Microbial Genomes (AAMG) pipeline^22^. AAMG was recently improved to handle metagenomic-scale data through supercomputing systems, such as Shaheen II (ranked no 7 in 2015 at https://www.top500.org/system/178515), available at King Abdullah University of Science and Technology (KAUST). GITs integrate annotations available from different sources, for every gene in a study as shown in Supplementary Figure, and here this format is recommended as a minimum standard for data integration, exploration and comparison of shotgun metagenomic samples. In order to expand the access to GITs and analytics of metagenomics data to larger scientific community, without the need of advanced computational skills or resources, we provide indexed GITs through KMAP’s online ‘Compare Module’ by extending and repurposing the standard framework of Metagenomic Reports (MetaRep^21^) software. See Supplementary Videos SV1 and SV2 on how to view and compare data sets in KMAP Compare Module. To contribute to the global effort of analyzing massive-scale microbial resources, we provide KMAP-based Gene Information Tables (GITs) from 40 gene catalogs. These GITs are based on ~ 3000 metagenomic samples capturing the comprehensive annotation of a global gene pool with over 275 million genes (see the Supplementary Table ST1 for links to GITs, POIs or set of annotations).

#### Comparison of KMAP features

Currently, several metagenomic data analysis pipelines offering a breadth of useful features already exists. A comparison of KMAP is performed with a few relevant platforms such as MG-RAST^23^, EBI Metagenomics^10^, Meta-Pipe^24^, MGX^25^, MetaRep^21^ and IMG/M^26^. Considering presence/absence and extent of implementation of different features categorized as types of input, gene prediction, gene annotation, visualization, comparison of samples and available computational resources, shows KMAP to be the most comprehensive platform thus far available (see Fig. 4).

The KMAP approach for improved analytics revolves around full-length genes from assembled genomes or metagenomes. Given a microbial genome, metagenomic contigs, or a gene catalog from any source as input, AAMG in the KMAP Annotation Module performs computationally expensive sequence comparisons against regularly updated reference databases. In the case of contigs, it provides a range of choices for gene prediction. Gene annotation is the main feature of annotation pipelines. There are a number of annotation features in KMAP not directly available in other systems, such as detection of Antibiotic Resistance Genes (ARG), Proteins of Interest with application to Industry (POIs) and Metagenomic Species (MS) binning using contigs as well as genes (see Supplementary Document on KMAP binning). AAMG integrates all the possible taxonomic and functional role assignments, including cross-references, to populate a GIT. In order to view, explore and compare metagenomic samples or gene catalogs, KMAP improves upon MetaRep and presents KMAP Compare Module by providing additional interactive heatmaps for more in-depth analysis of annotations, particularly automated estimation and comparative visualization of complete or incomplete KEGG pathway modules across selected datasets in one interactive figure (see Supplementary Video SV3). Additionally, in other systems like e.g. MG-RAST, it is not possible to look at both taxonomic and functional aspects using a single search. Here using GIT format, since we record complete information for a gene from different aspects, KMAP Compare Module allows the visualisation of results of a single query in the context of taxonomy, pathways, enzymes, KOs or GOs without re-issuing the search query every time.

### Exploring environmental functional genomics with KMAP

**Figure 5:**
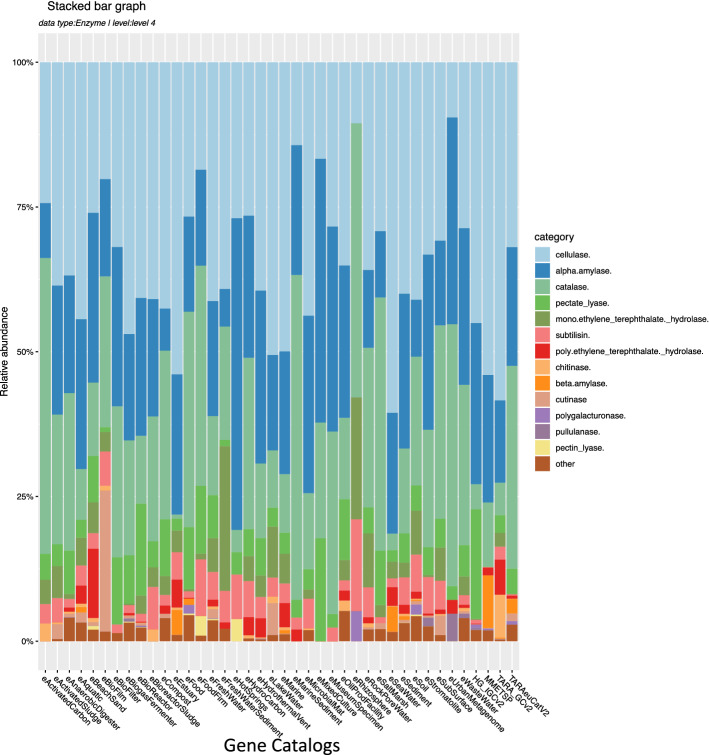
Examples of gene searches in KMAP. Relative abundance of selected extremozymes detected in a variety of microbial habitats. Supplementary Video SV3 shows how to navigate through KMAP to yield these results.

Gene Information Tables provided in this study can be used to lookup interesting genes based on specific identifiers for taxonomy, gene family ids e.g. KOs or Enzyme Classification (EC) numbers, GO ids or protein signature domain identifiers or preset “Filters”, using either commandline methods or online version of the GITs. KMAP Compare Module provides online access enabling larger audiences with minimal bioinformatics skills to mine interesting genes from datasets across different habitats. We demonstrate this utility of KMAP using two examples examining the distribution of (a) extremozymes and (b) antibiotic resistance genes (ARGs) across a range of contrasting habitats. Microbes from extreme environments are increasingly recognized as sources of novel compounds for biotechnological applications, with a potential of providing solutions for humanity’s great challenges, such as providing society with food, energy, and a clean environment^27,28^. Environmental metagenomics allows the exploration of previously inaccessible, genetic material from extreme environments that are likely, because of the challenges their extreme conditions pose to life, to contain extremozymes. Extremozymes can be very useful in industry, given their optimal activity and stability under extreme conditions (e.g. pressure, temperature and salinity), and can provide a foundation for developing environmentally friendly, efficient, and sustainable industrial technologies^28^. Most valuable cold and hot extremozymes include catalases, cellulases, proteases, lipases, mannases, pectinases and lacases^28^. We demonstrated the use of the KMAP “compare” module to explore using KMAP published metagenomes for the presence of enzymes of interest across a range of habitats, which showed microbial communities from some habitats to be enriched in these genes (Fig. 5).

Figure 5 shows the diversity of genes as percent of unique genes found in each of our range of selected habitats, revealing the general prevalence of cellulases over alpha-amylases, catalases, pectate lyases and hydrolases. For sequence search we provide a simple BLAST to create a list of related genes of interest from selected habitats, see online KMAP BLAST http://www.cbrc.kaust.edu.sa/kmapBLAST/, allowing download of hit sequences.

**Figure 6:**
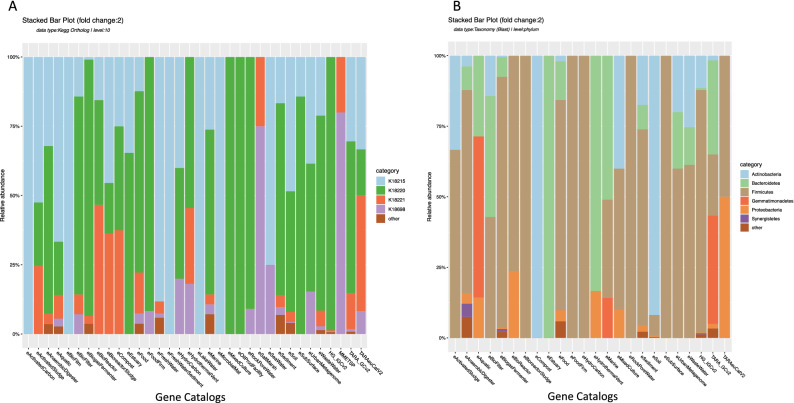
Antibiotic resistance genes. (**A**) Proportion of unique antibiotic resistance genes detected in different environments linked to tetracycline: tetV (K18215), tetM (K18220), tetX (K18221) and to beta lactamase class A: blaTEM (K18698). (**B**) Taxonomic affiliation of gene tetM in different habitats. See Supplementary Video SV4 to reproduce (**A**) in KMAP.

In another example, we look into the issue of antibiotic resistance by exploring the spread of recently reported top antibiotic resistance genes^29^ (ARGs) using tetX, tetM, and tetV (linked to tetracycline) as well as blaTEM (linked to beta lactamase class A) across different microbial habitats. Results in Fig. 6 show that tetracycline ARGs are prevalent across several environments. The taxonomic affiliation of unique genes for tetM (K18220), shows most of these genes are affiliated with Firmicutes in Human and several other habitats (Fig. 6B). However, different predominant affiliation for this gene were detected in specific environments: *Actinobacteria* in soil and compost metagenomes, *Bacteroidetes* in hydrothermal vents, estuaries and some other metagenomes, and *Gemmatimonadetes* in cold marine and aquatic metagenomes.

**Figure 7:**
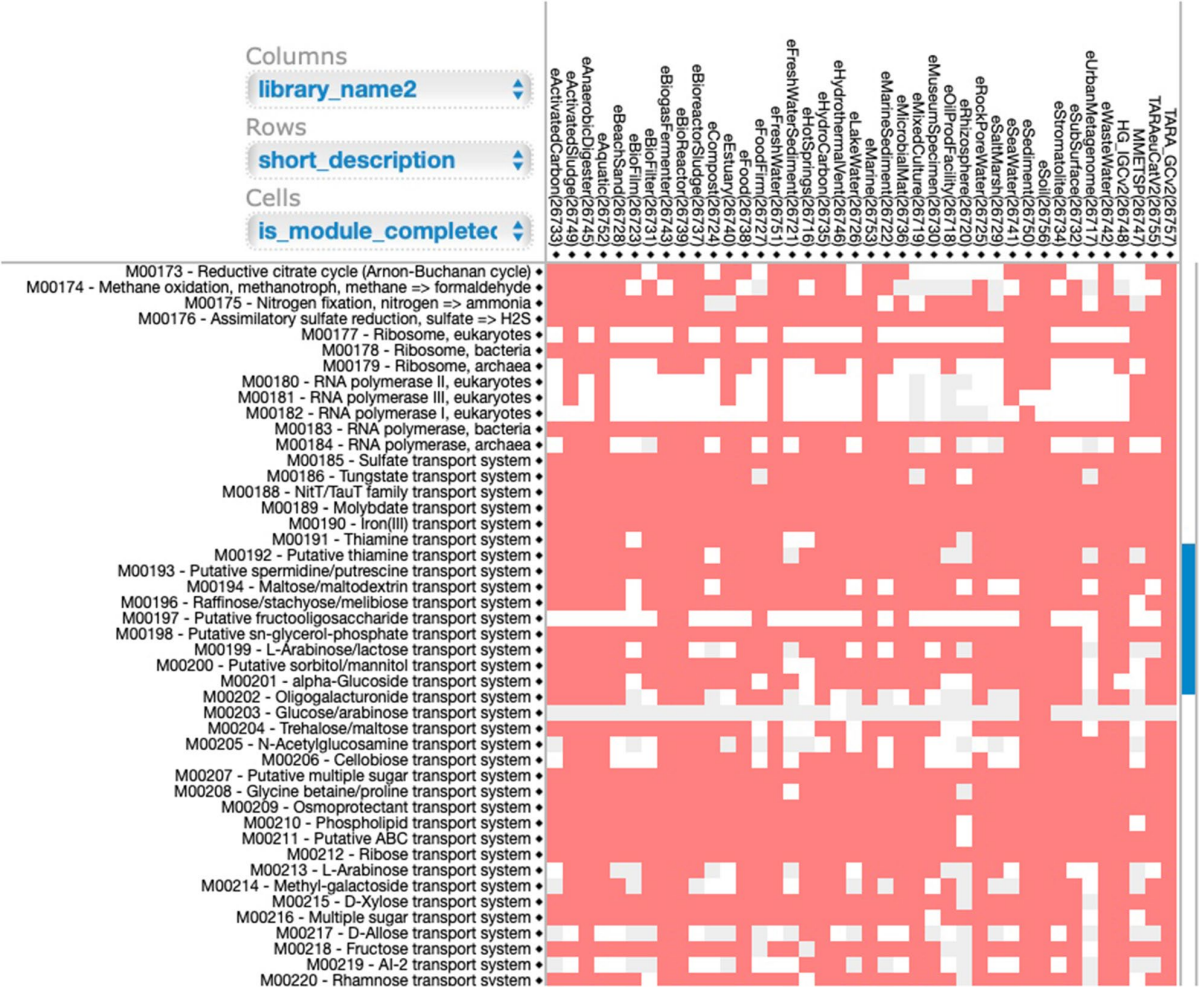
An interactive heatmap showing antibiotics resistance genes (ARGs) as complete (red color), incomplete (grey color) or not detected (white color) modules from KEGG KO pathways. A link to this interactive heatmap is available at https://bit.ly/2AHylwX, see Supplementary Video SV5 on how to reproduce Figure.

A unique feature of KMAP is the capability to present ‘filters’ (as shown in column 14 of the aforementioned GIT example), to focus on sets of genes with specific annotations. As an example, ARGs predicted via deepARG^30^ in KMAP are available through ‘filter:F.AntiBiotic.Resistance’. This ARG filter also includes ~ 30 classes of ARGs based on types of antibiotics, as provided by the deepARG reference database. Utilizing the ARG filter, (see methods query for ARGs), KMAP produces an interactive heatmap representing ARGs-related complete or incomplete KEGG Pathway modules across selected habitats (Fig. 7).

Clicking on a cell of this interactive heatmap provides more details and a link to KEGG module diagrams in order to examine how a module is shown to be complete or incomplete (Supplementary Video SV5). Since KMAP results from a query can also be saved as a table, these tables can be used to produce visualizations from any other system. An example heatmap using morpheus (https://software.broadinstitute.org/morpheus/), and providing individual ARG KOs and antibiotics across different habitats is shown at http://www.cbrc.kaust.edu.sa/aamg/habitats_KO_ARGs.svg. Here KO table obtained from KMAP using ARG filter query was appended with antibiotic types.

In general, all the gene catalogs presented in this study can easily be explored or compared using the KMAP Compare Module (see project number 119 with public access). However, this module suffers limitations when comparing large number of samples (e.g., above 50) due to the volume of data read-writes and Solr Lucene optimization compared to the available computer random access memory (RAM). These limitations can be addressed in future through optimizations for large-scale data visualization from platforms like Google Genomics^31^. We used servers with two terabytes of RAM. BLASTable version of our gene catalogs provide access to annotation and alignments through online BLAST based sequence comparisons, see Supplementary Video SV6 for an example.

## Discussion

Gene catalogs containing only full-length complete genes can be very useful for robust understanding of the taxonomic and functional repertoire of an environment and reliable research and development applications. For this purpose, long read sequencing technologies or assemblies of short reads are needed. After producing such gene catalogs, the annotation, analysis and comparison of metagenomic data sets is another challenge, particularly data integration. There are many existing tools and pipelines providing metagenomic data analysis, but each of these produce results using either a subset of tools, lack comparison functionality, or are not available online due to several limitations. Moreover, a comprehensive data integration is not possible due to different sets of standards or formats adopted by different annotation pipelines. For example, EBI metagenomics, now called MGnify, provides only InterPro based domains information for functional annotation of genes. MG-RAST provides annotation of gene function based on their own very specific but informative seed database. These and other similar pipelines comparing metagenomic sequences to public databases like UniProtKB and KEGG do not provide sequence comparison statistics for filtering the results based on user preferences. A common minimum standard for metagenomic data analysis can solve this data integration problem across different annotation platforms. The concept of GIT proposed here is very useful as it includes fields of identifiers for ontologies such as taxonomy, KOs, enzyme, Interpro, GO, COG, eggNOG. Similarly, it includes sequence comparison statistics (e.g. percent identity, percent coverage) tractable to user preferences. If different annotation pipelines are able to provide as much gene annotation information as possible but considering GIT like format, it can be useful for data exchange and comparison. For a community-based annotations initiative we provided here 27,000 assemblies of metagenomes from a variety of environments. Members of the Metagenomic community can perform annotations for any of these metagenomes using a platform of their liking and provide results e.g. in GIT like format. Once these GITs are considered as a standard, indexing these tables alongside the above-mentioned ontology reference databases, e.g. through the MetaRep^21^ framework, will allow to explore and compare metagenomic datasets at any selected level of ontology abstraction and such an indexing can provide fast access to these datasets online.

The advent of advanced metagenomics has accelerated discovery of microbial identification and function prediction, but has narrowed access of this capacity to scientists in nations with advanced bioinformatics and computational resources, widening the “genomics gap” between the capacity of developed and developing nations in exploring the potential applications of microbial functions^32,33^. KMAP provides equal access, by providing access to advanced KAUST computational resources and removing the requirement for advanced bioinformatics skills, to a wide community of researchers from across the world interested in exploring microbial communities and functions and having access to the internet.

## Conclusions

In essence, we provide here an online resource, KMAP, providing access to existing (re-annotated) and new gene catalogs, as a pilot study, from diverse environments. Inclusion of a few examples and screencast videos can help users without advanced computational skills and access to cutting-edge computational resources to explore the massive data for insights and comparison of taxonomic and functional repertoire of the biosphere, with options to filter data based on several parameters. Additionally, users can benefit from methods provided for short reads metagenomic sequence quality control, assembly and full length (compete) gene prediction for creation of non-redundant gene catalogs. As an ongoing commitment, the KMAP platform provides not only advanced analytics, but access to all publicly available shotgun metagenomic datasets, including ~ 27,000 metagenomic assemblies, which are being uploaded and made available through the KMAP platform.

More advanced users can benefit from the simple but comprehensive gene information tables. GITs from different microbial habitats and samples can provide a wealth of information for global ecosystem monitoring through machine learning and artificial intelligence efforts^34^. Central repositories with publicly accessible metadata for metagenomes, such as ENA, can easily integrate the gene information tables contributed by any research group. In turn, the broader scientific community can benefit from the predefined types of annotation in GITs, generated through KMAP or any other annotation platform, and work towards a deeper understanding of the untapped biochemical activities and functional capabilities of microbes in the biosphere to the benefit of humankind.

## Materials and methods

### Samples, assembly, full length gene predictions and creating gene catalogs for public shotgun metagenomes

We used the advanced search function at EBI to create a list of fastq files for metagenomes whose taxonomy was restricted to metagenomes and shotgun sequencing platform was restricted to paired-end Illumina sequencing technology. Resulting metadata file was filtered for availability of ftp location to download fastq files. We downloaded ~ 27,000 metagenomes using wget and GridFTP, while keeping track of the ENA run, sample, project, and metagenome taxon identifiers. Upon download, we pre-processed the individual samples for a quality control and validation of the pairs using bbduk (http://jgi.doe.gov/data-and-tools/bb-tools/). Computationally demanding assembly was performed using MegaHit assembler^35^ (final contig size limited to 500 bp) with default options, at KAUST supercomputing resources. In this pilot study, to produce example gene catalogs, we selected a subset of ecological metagenomes (as defined in ENA metagenome types) and performed predictions of the complete genes using Prodigal^17^ (maintaining a minimum length of 100 bp) with an option to restrict gene prediction to complete genes only. To create gene catalogs based on complete genes, we clustered these genes from each environment using CD-HIT to produce a nonredundant gene catalogs, keeping percent identity to 90, coverage percent to 95 and length difference to 80.

### Annotation of gene catalogs and Gene Information Tables (GITs)

The gene catalogs were annotated using BLAST based comparison to Universal Proteins Knowledgebase (UniProtKB, www.uniprot.org) to taxonomic, COGs and eggNOG ontology assignments. Similarly, KEGG (www.kegg.jp) sequences with a assigned KEGG Ortholog (KO) gene family identifier were compared to obtain annotation of function and enzymes. InterPro database was used to obtain function signature domains and gene ontology (GO) information. Annotation results from individual searches against multiple sources were combined into an extended Gene Information Table (GIT) format containing taxonomic and functional identifiers, sources, blast similarity statistics and other filters. These annotation methods are implemented into the extended version of Automatic Annotation of Microbial Genomes (AAMG) pipeline, now available as Annotation Module through KAUST Metagenomic Analysis Platform (KMAP). Gene information tables from gene catalogs reported in this study were indexed and deposited to KMAP Compare Module, project number 119.

Genes originating from individual samples are clustered together to produce a common reference gene catalog. Mapping reads from individual samples onto the gene catalog provides a table of unique genes across samples, where each cell represents a gene abundance of zero or more for its corresponding sample. Such a table allows comparisons with a degree of enrichment by showing which genes are unique or common across which samples. Adding another dimension of gene annotation information to this table provides the potential taxonomic and functional roles of the genes in this catalog. We perform indexing of complete gene information tables from each gene catalog for exploration through the Compare Module. Similarly, sample-specific gene information tables are obtained from a relevant gene catalog based on the gene abundance information available for a sample. Gene abundance tables were already available for 243 samples for the Tara study and 1267 samples for the Human Gut Microbiome. We produced gene information tables for these gene catalogs alongside MMETSP and Tara micro-eukaryotic gene catalogs. Then, we performed Solr Lucene indexing and deposition of these gene catalogs to KMAP project number 119. We also produced and indexed sample groups by combining related samples from Tara or Human Gut Microbiome (see the *KMAP documentation* for more details).

Statistical analysis was done in R programming environment. Alpha diversity estimates comparing original annotation and the re-annotated Tara Ocean and HGM gene catalogs was done with the “vegan” package v2.5-7 (https://github.com/vegandevs/vegan).

### KAUST Metagenomic Analysis Platform (KMAP) interface

KMAP interface main webpage (https://www.cbrc.kaust.edu.sa/aamg/kmap.start/) serves as a central point allowing access to KMAP modules, Documentation, Supplementary Data 1, examples through screencast videos and access to metagenomic assemblies as well as downloading of annotations for gene catalogs available in this study. Access to KMAP database for browsing and comparing gene catalogs is provided through ‘Try KMAP’ or the direct ‘database’ link on the main page without any requirement for login. For users who wish to keep their data saved for a while can log in and submit contigs, gene catalogs (DNA or protein data) for annotation. KMAP database uses an existing framework called Metagenomic Reports (MetaRep^21^), a Php based web application that allows comparison of metagenomic data. We improved and expanded this application to include additional visualizations and our metagenomic annotation module. The following sections describe KMAP annotation and compare modules.

### KMAP Annotation Module

The Annotation Module of the KMAP is an improved version of our annotation pipeline AAMG^22^, see Fig. 4A. Using KAUST’s supercomputing facilities, this component processes input sequences and performs sequence comparisons at a user-defined BLAST alignment bitscore cutoff value (default: 50). All sequence comparisons are processed in parallel by splitting the input sequence data into 2 Mb chunks. These comparisons are performed against UniProtKB based on the best hit and least common ancestor (LCA) for the taxonomic assignments, and against KEGG sequences having KEGG Orthology (KO) for the robust functional role assignments. Cross-references to eggNOG are retrieved using UniProtKB hits. However, enzyme, module, and pathway cross-references are obtained from KO. An analysis by InterProScan is performed to derive the Gene Ontology (GO) and likely functional signature domains. AAMG provides cross-references to our curated datasets of bioactive genes, as well as the dataset of enzymes and POIs. As shown in Fig. 1B, AAMG performs additional RNA predictions and annotations when assembled contigs are the input. At the annotation integration stage, a gene information AAMG TSV table is produced by applying additional BLAST cutoffs such as percent identity of 30 and a percent query coverage of 50. The complete output from AAMG is saved on a web server, providing downloads and visualizations for several annotation categories. Additional options for the taxonomic binning of contigs or genes and COGs binning is available for each project at the AAMG output webpage. Supplementary Table ST1 shows universal resource locations (URLs) for every dataset annotated using AAMG in this study. Details on KMAP annotation procedure and references to the resources mentioned in this section are provided in the *KMAP documentation*.

### KMAP compare module

KMAP compare module is an extension of MetaRep^21^ framework, the standard version of this framework provides browsing, searching and comparison of available datasets. It requires to setup a mysql database of reference ontologies such as ncbi taxonomy, KEGG, Gene Ontology, ezymes, InterPro, etc. to present results on user selected ontology levels. KMAP Annotation Module based GITs provide relevant identifiers and BLAST statistics to index complete annotation of a metagenomic sample or a gene catalog. We implemented parallel indexing of GITs from multiple samples or gene catalogs. One of the exciting new visualizations in the Compare Module is a clickable heatmap that enables the exploration of either complete or incomplete KEGG pathway modules across a selected set of samples. An example is shown in Fig. 7. Additional interactive heatmaps are provided to pinpoint the magnitude of fold changes from sample comparisons for a selected annotation category. KMAP documentation provides a complete walk through with examples starting from the use of annotation module leading to example analysis and comparisons available in the Compare Module.

## Supplementary Information

Supplementary Information 1.

Supplementary Information 2.

Supplementary Information 3.

Supplementary Information 4.

Supplementary Information 5.

Supplementary Information 6.

## Data Availability

Data presented in this work is available through http://www.cbrc.kaust.edu.sa/aamg/kmap.start, and ENA meta-project accession PRJEB31567.
