# Publisher Correction: KAUST Metagenomic Analysis Platform (KMAP), enabling access to massive analytics of re-annotated metagenomic data

**DOI:** 10.1038/s41598-021-93386-3

**Published:** 2021-07-01

**Authors:** Intikhab Alam, Allan Anthony Kamau, David Kamanda Ngugi, Takashi Gojobori, Carlos M. Duarte, Vladimir B. Bajic

**Affiliations:** 1 Computational Bioscience Research Center, King Abdullah University of Science and Technology (KAUST), Thuwal, 23955-6900 Saudi Arabia; 2 Leibniz Institute DSMZ-German Collection of Microorganisms and Cell Cultures, Inhoffenstraße 7B, 38124 Brunswick, Germany; 3 Red Sea Bioscience Research Center, King Abdullah University of Science and Technology (KAUST), Thuwal, 23955-6900 Saudi Arabia

Correction to: *Scientific Reports* 10.1038/s41598-021-90799-y, published online 01 June 2021

The original version of this Article contained errors.

In the Abstract,

“However, this also generates a procedural bottleneck for on-going re-analysis as reference databases grow and methods improve, and analyses need be updated for consistency, which require acceess to increasingly demanding bioinformatic and computational resources.”

now reads:

“However, this also generates a procedural bottleneck for on-going re-analysis as reference databases grow and methods improve, and analyses need be updated for consistency, which require access to increasingly demanding bioinformatic and computational resources.”

Additionally, Figure 3 was distorted. The original Figure 3 and accompanying legend appear below.

**Figure 3:**
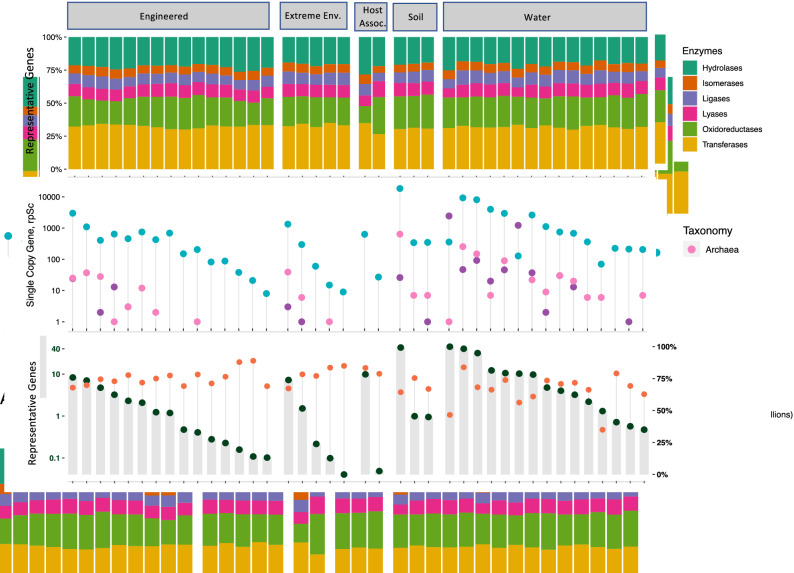
Gene catalogs showing count of habitat specific representative genes. (**A**) The count of total representative genes (million) as well as annotated genes (percent). (**B**) The count of taxon assigned single copy marker gene rpSc (PF01738) is shown on the log scale and (**C**) unique genes labeled with enzymes of different types. This data presents 36 new gene catalogs from ecological metagenomes, marked with initial “e”. Existing four gene catalogs are also included alongside the global metagenomic gene catalog, see Supplementary Table S1 for details on sample count, study accessions and corresponding annotation from KAMP.

Furthermore, a sentence was omitted from the Acknowledgements section.

“We are thankful to our KAUST Supercomputing Laboratory (KSL) and KAUST High Performance Computing (HPC) laboratory for providing continued support and access to the required computer resources. We are thankful to Aleksandar Radovanovic for help in developing a webpage for KMAP and Tahira Jamil for help with producing one of the figures and Mahmut Uludag for the biom format utility.”

now reads:

“This work is dedicated to late Vladimir B. Bajic (https://www.ncbi.nlm.nih.gov/pmc/articles/PMC7056845/) for his vision and leading KMAP project. We are thankful to our KAUST Supercomputing Laboratory (KSL) and KAUST High Performance Computing (HPC) laboratory for providing continued support and access to the required computer resources. We are thankful to Aleksandar Radovanovic for help in developing a webpage for KMAP and Tahira Jamil for help with producing one of the figures and Mahmut Uludag for the biom format utility.”

Finally, author Vladimir B. Bajic is deceased.

The original Article has been corrected.

