# Supplementary material for: KAUST Metagenomic Analysis Platform (KMAP), enabling access to massive analytics of re-annotated metagenomic data
Source: Sci Rep. 2021 Jun 1;11:11511. doi: 10.1038/s41598-021-90799-y (PMC8169707; doi:10.1038/s41598-021-90799-y)
Supplement: Supplementary file 1 — Supplementary Information 1. [file 41598_2021_90799_MOESM1_ESM.pdf]

Supplementary Information File.

KAUST Metagenomic Analysis Platform (KMAP), enabling access to massive analytics of re-annotated metagenomic data.

Authors

Intikhab Alam[1*], Allan Anthony Kamau[1], David Kamanda Ngugi[2], Takashi Gojobori[1], Carlos M. Duarte[1,3] and Vladimir B. Bajic[1].

Supplementary Materials

Supplementary website: https://www.cbrc.kaust.edu.sa/aamg/KMAP_Data/

Supplementary Table 1, Gene Catalogs, ENA Project Ids, Links to GITs and KMAP Annotations.
Supplementary Table 2, ENA Studies and Samples.
Supplementary Table 3, ~27000 Metagenome Assembly Stats.
Supplementary Figures, Description of SF0. GIT and SF1. Gene Catalog groups.
Supplementary methods, include linux commandlines and database queries.
Supplementary Binning Document.
Supplementary KMAPtsv2Biome.Example.tgz, KMAP GIT format into biom format (requires biom module), see supplementary methods..
Supplementary KMAP Documentation.

KMAP screencast videos.
SV1. Introduction. How to View/Browse data in KMAP:
KMAP_Intro_ViewData1.mp4

SV2. How to Compare data in KMAP:
KMAP_Intro_CompareData1.mp4

SV3. How to search interesting enzymes (e.g. extremozymes) in KMAP:
KMAP_Extremozymes_Query_v1.mp4

SV4. How to Search Antibiotic Resistance Genes (ARGs) in different microbial habitats in KMAP
KMAP_Example_ARGs_in_habitats_v1.mp4

SV5. How to make/use interactive heatmaps (e.g. pathway modules) in KMAP:
KMAP_Interactive_heatmap_ARGs_in_habitats_v1.mp4

SV6. How to BLAST a sequence in KMAP Global Proteome:
KMAP_PETase_BLAST_in_GlobalMetaProteome.mp4
