# Supplementary material for: KAUST Metagenomic Analysis Platform (KMAP), enabling access to massive analytics of re-annotated metagenomic data
Source: Sci Rep. 2021 Jun 1;11:11511. doi: 10.1038/s41598-021-90799-y (PMC8169707; doi:10.1038/s41598-021-90799-y)
Supplement: Supplementary file 2 — Supplementary Information 2. [file 41598_2021_90799_MOESM2_ESM.pdf]

## KMAP Metagenomic binning using contigs or genes

One of the unique features of KMAP's annotation module is to provide putative Metagenomic Species (MS) binning based on contigs (when input used is contigs) or genes (when input used is CDSes or proteins). This grouping or binning is achieved by summarizing taxonomic information from the majority of genes to contigs using either the Best Hit (BH) or the lowest-common-ancestor (LCA) approach. Resulting bins are subjected to bin completion and contamination validation based on 40 universal single copy genes. In addition a bin refiner such as DAS_Tool (Sieber et al., 2018) is used to independently verify and pick most complete and less contaminated bins from the list of bins available in KMAP from phylum to species level. A benchmarking of the binning approach implemented in KMAP and other state-of-the-art binning programs based on a synthetic metagenomic community from the CAMI Experiment (Sczyrba et al., 2017) encompassing low, medium and high complexity microbiomes (Figures 3 A, B and C, respectively), reveals that integrated binning approaches (Sieber et al., 2018) are much better than individual methods and also showcase the utility of the combined-binning approach implemented in KMAP.

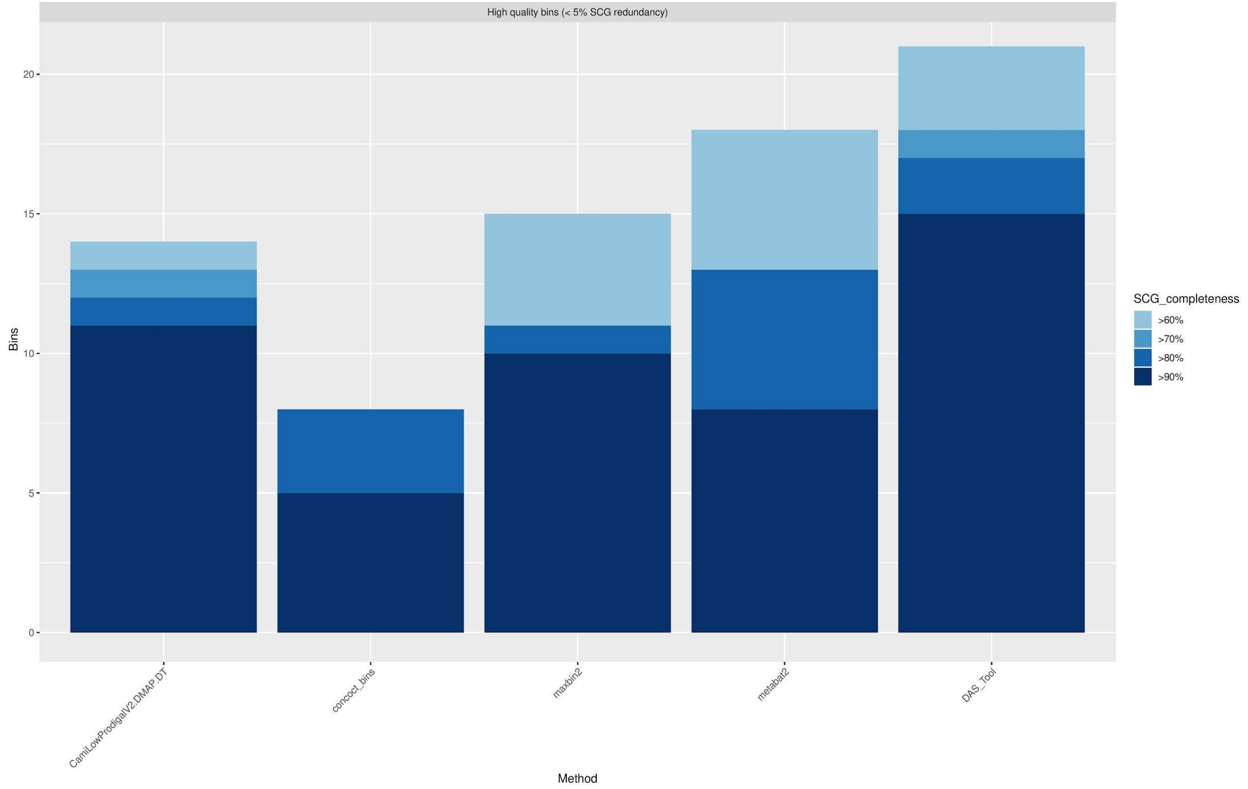


Figure 1A. Contig binning comparison for KMAP vs other methods using CAMI Low complexity data

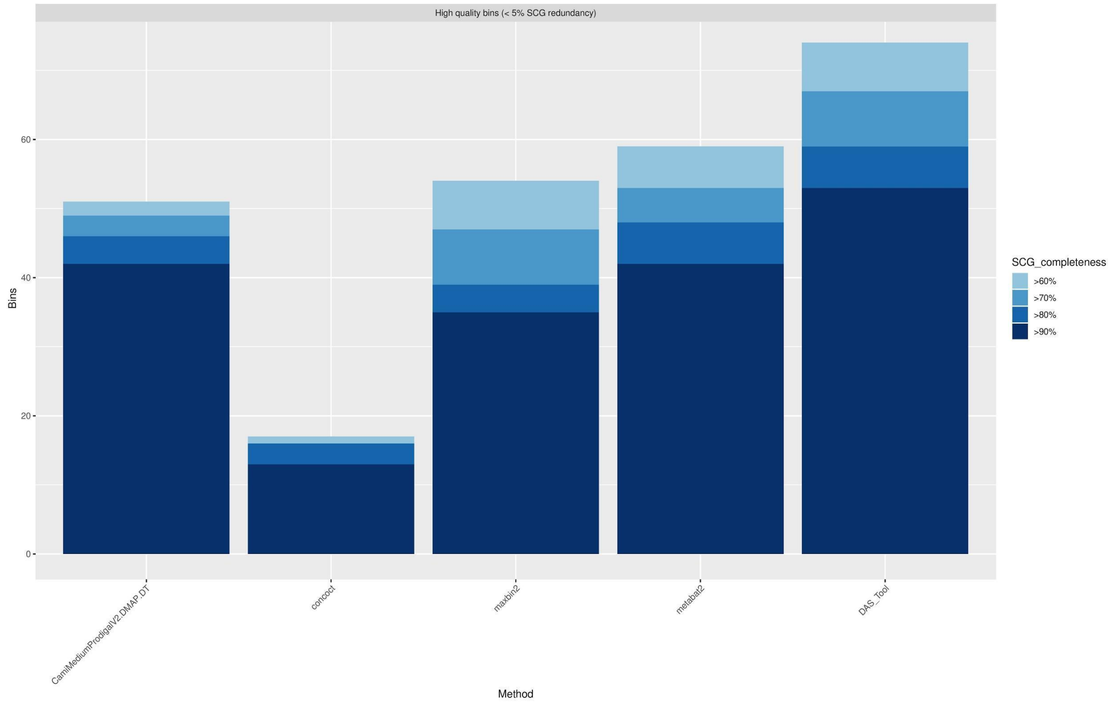


Figure 1B. Contig binning comparison for KMAP vs other methods using CAMI Medium complexity data

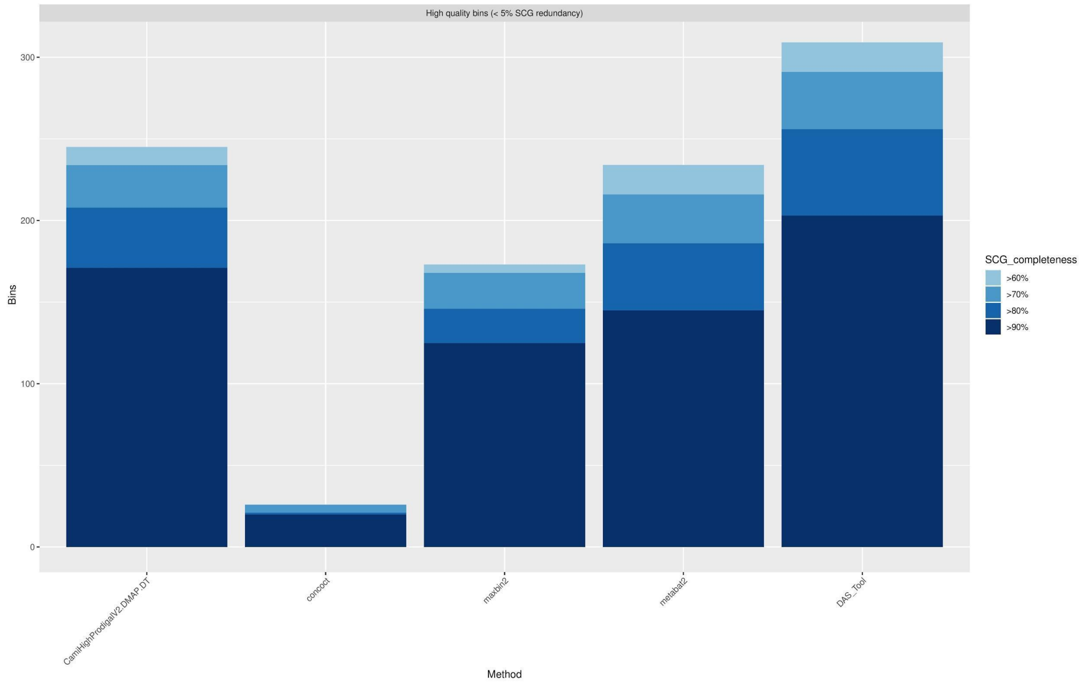


Figure 1C. Contig binning comparison for KMAP vs other methods using the CAMI High complexity microbiome data

We also compared KMAP binning results using either contigs or gene catalogs as input (Figure 2). Performance appears to be very close; however, with increasing species complexity (more closer species included as in medium and high complexity CAMI data sets), number of bins are reduced due to increasing number of genes masked behind the non-redundant representative genes encompassing the gene catalogs. KMAP provides all putative MS bins to be explored on individual phylum to species taxonomic levels, with either BH or LCA approach. DAS_Tool results are also provided for every project that is annotated via KMAP Compare Module.

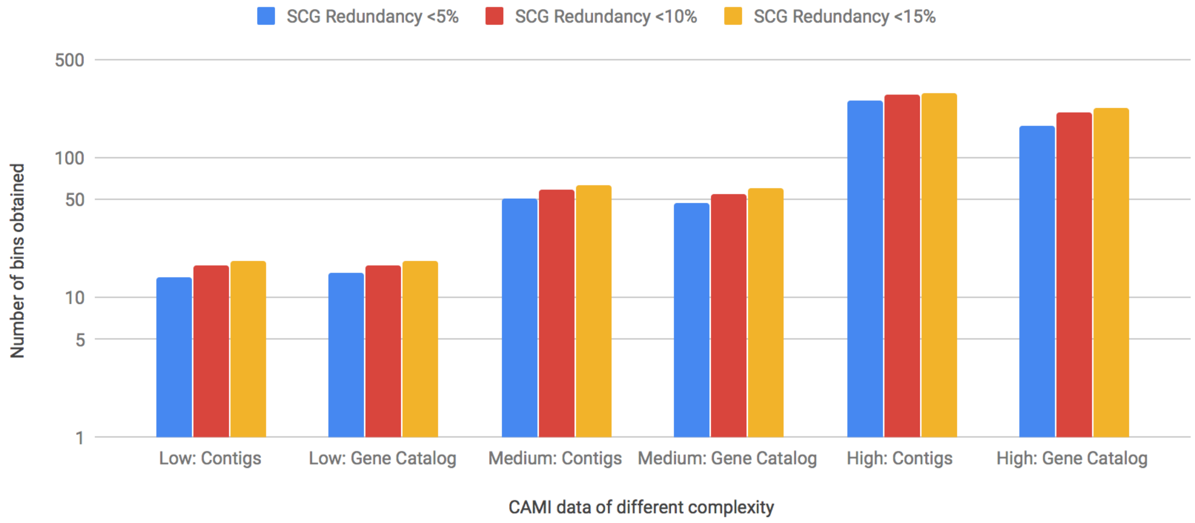


Figure 2. KMAP contig binning vs gene catalog binning using CAMI data at different levels of species complexity.

References:

Sieber, C.M., Probst, A.J., Sharrar, A., Thomas, B.C., Hess, M., Tringe, S.G. and Banfield, J.F., 2018. Recovery of genomes from metagenomes via a dereplication, aggregation and scoring strategy. *Nature microbiology*, *3*(7), p.836.

Sczyrba, A., Hofmann, P., Belmann, P., Koslicki, D., Janssen, S., Dröge, J., Gregor, I., Majda, S., Fiedler, J., Dahms, E. and Bremges, A., 2017. Critical assessment of metagenome interpretation—a benchmark of metagenomics software. *Nature methods*, *14*(11), p.1063.
