# Supplementary material for: KAUST Metagenomic Analysis Platform (KMAP), enabling access to massive analytics of re-annotated metagenomic data
Source: Sci Rep. 2021 Jun 1;11:11511. doi: 10.1038/s41598-021-90799-y (PMC8169707; doi:10.1038/s41598-021-90799-y)
Supplement: Supplementary file 3 — Supplementary Information 3. [file 41598_2021_90799_MOESM3_ESM.pdf]

Supplementary Table. List of new and existing metagenomic gene catalogues (GCs) annotated using AAMG pipeline in KMAP.									
**#**	**KMAP GC Name**	**ENA TaxonID_MetagenomeType**	**Samples Count**	**ENA Study IDs**	**Genes Count**	**% Annotated**	**Gene Info Table**	**Industrial POIs**	**AAMG Results**
1	eAquatic	1169740_aquatic.metagenome	79	PRJEB14197, PRJEB22997, PRJNA214436, PRJNA327989, PRJNA377521	9744953	61.00	TSV	POIs	AAMG WWW Output
2	eEstuary	1649191_estuary.metagenome	36	PRJNA312987, PRJNA320136	3224006	71.82	TSV	POIs	AAMG WWW Output
3	eFreshWater	449393_freshwater.metagenome	76	PRJEB23036, PRJEB9136, PRJNA104935, PRJNA214105, PRJNA254927, PRJNA259931, PRJNA261364, PRJNA282166, PRJNA312830, PRJNA312985, PRJNA312986, PRJNA342151, PRJNA64033, PRJNA64035, PRJNA64037, PRJNA64039, PRJNA64041	12253025	66.32	TSV	POIs	AAMG WWW Output
4	eFreshWaterSediment	556182_freshwater.sediment.metagenome	24	PRJEB14421, SRP001573	474067	62.85	TSV	POIs	AAMG WWW Output
5	eLakeWater	1647806_lake.water.metagenome	2	PRJEB6029	570675	69.31	TSV	POIs	AAMG WWW Output
6	eMarine	408172_marine.metagenome	398	PRJDB4437, PRJDB5777, PRJDB5792, PRJEB11384, PRJEB12379, PRJEB14127, PRJEB14154, PRJEB14899, PRJEB19456, PRJEB87662, PRJEB9136, PRJNA248552, PRJNA249576, PRJNA249643, PRJNA257723, PRJNA266633, PRJNA266672, PRJNA266673, PRJNA266674, PRJNA266676, PRJNA266677, PRJNA266678, PRJNA266679, PRJNA266685, PRJNA266687, PRJNA266688, PRJNA277705, PRJNA278075, PRJNA291283, PRJNA291491, PRJNA316064, PRJNA318731, PRJNA352798	31669846	68.27	TSV	POIs	AAMG WWW Output
7	eMarineSediment	412755_marine.sediment.metagenome	3	PRJNA259156	1314237	34.99	TSV	POIs	AAMG WWW Output
8	eMicrobialMat	527640_microbial.mat.metagenome	7	PRJEB14856, PRJEB25599, PRJNA283228	2196091	66.30	TSV	POIs	AAMG WWW Output
9	eRockPoreWater	1531857_rock.porewater.metagenome	10	PRJNA257561	715243	79.11	TSV	POIs	AAMG WWW Output
10	eSeaWater	1561972_seawater.metagenome	29	PRJEB12234, PRJEB14154, PRJNA272679, PRJNA302103, PRJNA319949	4012972	70.92	TSV	POIs	AAMG WWW Output
11	eSediment	412755_marine.sediment.metagenome	3	PRJNA259156	10559106	73.94	TSV	POIs	AAMG WWW Output
12	eWasteWater	527639_wastewater.metagenome	114	PRJEB10139, PRJEB13832, PRJEB13978, PRJEB6461, PRJEB7844, PRJEB7850, PRJEB8669, PRJNA230567, PRJNA244282, PRJNA264280, PRJNA289616, PRJNA294040	4776059	73.56	TSV	POIs	AAMG WWW Output
13	eBeachSand	412757_beach.sand.metagenome	16	PRJNA260285	949014	66.94	TSV	POIs	AAMG WWW Output

14	eSoil	410658_soil.metagenome	164	PRJEB10291, PRJEB10725, PRJEB12864, PRJEB21930, PRJEB5122, PRJEB6384, PRJEB7626, PRJEB9798, PRJNA186499, PRJNA205039, PRJNA208116, PRJNA225837, PRJNA236429, PRJNA239941, PRJNA281298, PRJNA287178, PRJNA287582, PRJNA313136, PRJNA384362, SRP011419	42964902	64.44	TSV	POIs	AAMG WWW Output
15	eSubSurface	1564682_subsurface.metagenome	7	PRJNA276301, PRJNA276302, PRJNA276303, PRJNA276304, PRJNA276305, PRJNA276306	991828	75.48	TSV	POIs	AAMG WWW Output
16	eRhizosphere	939928_rhizosphere.metagenome	8	PRJDB5626	48771	78.82	TSV	POIs	AAMG WWW Output
17	eHotSprings	433727_hot.springs.metagenome	3	PRJEB7848, PRJNA291689, PRJNA294671	40388	85.00	TSV	POIs	AAMG WWW Output
18	eHydroCarbon	938273_hydrocarbon.metagenome	4	PRJNA183510, PRJNA291107	1518934	78.38	TSV	POIs	AAMG WWW Output
19	eHydrothermalVent	652676_hydrothermal.vent.metagenome	24	PRJEB11362, PRJEB15554, PRJEB19456, PRJNA234377, PRJNA290104, PRJNA312413	7232878	67.34	TSV	POIs	AAMG WWW Output
20	eOilProdFacility	885331_oil.production.facility.metagenome	3	PRJNA276694	98984	83.62	TSV	POIs	AAMG WWW Output
21	eSaltMarsh	1504975_salt.marsh.metagenome	6	PRJEB7769	216338	77.13	TSV	POIs	AAMG WWW Output
22	eActivatedCarbon	1214127_activated.carbon.metagenome	8	PRJNA301005	1185300	77.47	TSV	POIs	AAMG WWW Output
23	eActivatedSludge	942017_activated.sludge.metagenome	20	PRJDB4244, PRJEB11413, PRJNA194095, PRJNA219165, PRJNA226634, PRJNA236782, PRJNA295114	8316278	67.96	TSV	POIs	AAMG WWW Output
24	eAnaerobicDigester	1263854_anaerobic.digester.metagenome	28	PRJEB10932, PRJEB20855, PRJEB22104, PRJNA283612	7028643	69.89	TSV	POIs	AAMG WWW Output
25	eBioFilm	718308_biofilm.metagenome	6	PRJNA189999, PRJNA295849, PRJNA299404	408163	78.26	TSV	POIs	AAMG WWW Output
26	eBioFilter	1070537_biofilter.metagenome	1	PRJNA202074	279490	71.29	TSV	POIs	AAMG WWW Output
27	eBiogasFermenter	718289_biogas.fermenter.metagenome	25	PRJDB5067, PRJEB21266, PRJEB8813, PRJNA311376, PRJNA311377, PRJNA311378, PRJNA311379, PRJNA311380, PRJNA311381, PRJNA311382, PRJNA311383	4738837	74.56	TSV	POIs	AAMG WWW Output
28	eBioReactor	1076179_bioreactor.metagenome	20	PRJDB4244, PRJEB12797, PRJNA158439, PRJNA209200, PRJNA226580, PRJNA279279, PRJNA81811	3227874	72.96	TSV	POIs	AAMG WWW Output
29	eBioreactorSludge	412754_bioreactor.sludge.metagenome	11	PRJNA171369, PRJNA231881, PRJNA254356, PRJNA282558	2080835	71.94	TSV	POIs	AAMG WWW Output
30	eCompost	702656_compost.metagenome	2	PRJEB14766	475795	69.21	TSV	POIs	AAMG WWW Output
31	eFood	870726_food.metagenome	12	PRJNA286900, PRJNA327808	2309726	77.77	TSV	POIs	AAMG WWW Output
32	eFoodFirm	1154581_food.fermentation.metagenome	6	PRJEB19846, PRJNA288044	158401	87.95	TSV	POIs	AAMG WWW Output
33	eMixedCulture	1306859_mixed.culture.metagenome	8	PRJNA288044	108603	88.99	TSV	POIs	AAMG WWW Output
34	eMuseumSpecimen	1661029_museum.specimen.metagenome	2	PRJDB4067	228059	76.53	TSV	POIs	AAMG WWW Output

35	eStromatolite	496921_stromatolite.metagenome	2	PRJNA315555, PRJNA317551	1235798	75.09	TSV	POIs	AAMG WWW Output
36	eUrbanMetagenome	1591089_urban.metagenome	24	PRJNA301589	102147	69.12	TSV	POIs	AAMG WWW Output
37	TARA_GCv2	NA	243	NA	40154704	83.91	TSV	POIs	AAMG WWW Output
38	HG_IGCv2	NA	1267	NA	9879502	83.40	TSV	POIs	AAMG WWW Output
39	CamiLowProdigalV2	NA	1	NA	140385	93.77	TSV	POIs	AAMG WWW Output
40	CamiMediumProdigalV2	NA	2	NA	452003	96.35	TSV	POIs	AAMG WWW Output
41	CamiHighProdigalV2	NA	5	NA	1943423	97.67	TSV	POIs	AAMG WWW Output
42	MESOCOSMv2	NA	65	NA	2776201	63.85	TSV	POIs	AAMG WWW Output
43	TARAeuCatV2	NA	441	NA	44972470	46.65	TSV	POIs	AAMG WWW Output
44	MMETSP	NA	678	NA	10257897	56.32	TSV	POIs	AAMG WWW Output
		Total Samples	3893		278032851	73.33			
		Samples in previous gene catalogs	2702						
		samples in new gene catalogs	1191						
