# Supplementary material for: KAUST Metagenomic Analysis Platform (KMAP), enabling access to massive analytics of re-annotated metagenomic data
Source: Sci Rep. 2021 Jun 1;11:11511. doi: 10.1038/s41598-021-90799-y (PMC8169707; doi:10.1038/s41598-021-90799-y)
Supplement: Supplementary file 5 — Supplementary Information 5. [file 41598_2021_90799_MOESM5_ESM.pdf]

Supplementary Figures

Column #	Category	Example
1	Gene ID	CamiLowProdigal_000000003
2	Sample ID	CamiLowProdigal
3	Gene Name	two-component system, OmpR family, phosphate regulon sensor histidine kinase PhoR [EC:2.7.13.3]
4	Gene Sources	contig:RL_S1_C2:i:3:1061-1954_- KEGG:prs:B9H02_07610 +InterPro:His Kinase A...
5	GO ID	GO:0000155||GO:0007165
6	GO Source	PF00512
7	EC ID	2.7.13.3
8	EC Source	KEGG:prs:B9H02_07610
9	InterPro ID	PF00512||PF02518
10	Taxon ID	1120996
11	Evalue	0
12	Percent Identity	100
13	Percent Coverage	100
14	Filters	B.KO||C.Enzyme||E.COG||B.UniprotKB||D.InterPro||A.Annotated
15	KO ID	K07636
16	KO Source	KEGG:prs:B9H02_07610
17	Weight	read counts ...
18	COG ID	COG0642
19	eggNOG ID	eggNOG.ID

Figure S0. An example of Gene Information Table, here columns are shown as rows.

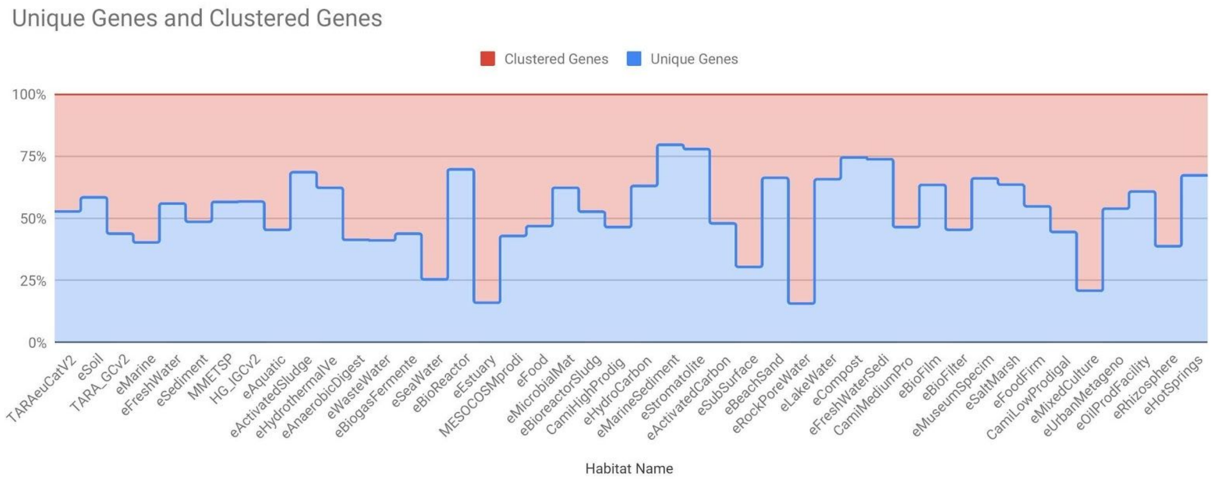

Figure S1. Percent of habitat specific non-redundant genes appear globally unique or common across different habitats
