# Supplementary material for: KAUST Metagenomic Analysis Platform (KMAP), enabling access to massive analytics of re-annotated metagenomic data
Source: Sci Rep. 2021 Jun 1;11:11511. doi: 10.1038/s41598-021-90799-y (PMC8169707; doi:10.1038/s41598-021-90799-y)
Supplement: Supplementary file 6 — Supplementary Information 6. [file 41598_2021_90799_MOESM6_ESM.pdf]

KMAP Supplementary Document
Samples, assembly, full length gene predictions and creating gene catalogs for public shotgun metagenomes

We used the advanced search function at EBI to create a list of fastq files for metagenomes whose taxonomy was restricted to metagenomes and shotgun sequencing platform was restricted to paired-end Illumina sequencing technology, as shown in the following query:

```
tax_tree(408169) AND library_layout="PAIRED" AND instrument_platform="ILLUMINA" AND library_strategy="WGS" AND library_source="METAGENOMIC" AND nominal_length>=100 AND base_count>=200000000
```

Resulting metadata file was filtered for availability of ftp location to download fastq files. We downloaded ~27000 metagenomes using wget and GridFTP, while keeping track of the ENA run, sample, project, and metagenome taxon identifiers. Upon download, we pre-processed the individual samples for a quality control and validation of the pairs using bbduk (http://jgi.doe.gov/data-and-tools/bb-tools/), as shown in the following commandlines:

Read cleaning using Bbduk:

```
$bbduk in=$r1 in2=$r2 overwrite=true prealloc=t k=23 ktrim=r mink=11 hdist=1 qtrim=rl trimq=10
minlength=60 threads=4 ref=adapters.fa,phix174_ill.ref.fa.gz tbo tpe |$repair.sh in=stdin.fq
out=$r1.trim.filt_1.fq.gz out2=$r2.trim.filt_2.fq.gz outs=$r.single.fq.gz overwrite=true"
```

Computationally demanding assembly was performed using MegaHit assembler (34) (final contig size limited to 500 bp) with default options, at KAUST supercomputing resources, as shown in the commandline below:

Metagenomic assembly using Megahit:

```
$megahit -1 $r1.trim.filt_1.fq.gz -2 $r1.trim.filt_2.fq.gz --continue $ksteps -t 64 --min-contig-
len 500 --out-prefix $sample.min500 -o $assemblyDir/$sample.megahit
```

In the first phase, to produce example gene catalogs, we selected ecological metagenomes only and performed predictions of the complete genes using Prodigal (17) (maintaining a minimum length of 100 bp), as shown in the following commandline:

Gene prediction (complete genes) using Prodigal:

```
prodigal -c -p m -i $infile -a $outdir/$id\_prodigal.aa -d $outdir/$id\_prodigal.fna -f gff -o
$outdir/$id\_prodigal.gff -m -q
```

Creating Gene Catalogs

We clustered these genes using CD-HIT to produce a nonredundant set called a gene catalog, one for each type of ecological metagenome, keeping percent identity to 90, coverage percent to 95 and length difference to 80, as shown in the following commandline:

Gene clustering (gene catalog) using CD-HIT:

```
cd-hit-est -i $in.fna -o $out.fna -c 0.95 -T 32 -M 0 -G 0 -aS 0.9 -g 1 -r 1 -d 0 -s 0.8
```

Reads mapping to gene catalogs using Bbmap:

```
$bbmap threads=8 in=$r1.trim.filt_1.fq.gz in2=$r1.trim.filt_2.fq.gz ref=$geneCatalog nodisk
rpkm=$fpkm ambig=toss idfilter=0.9 tossbrokenreads
```

Following are a few queries used in KMAP compare module to search interesting gene sets in microbial gene catalogs available in KMAP compare module

Enzymes search query in KMAP Compare Module:

The following query can be used to find extremozymes in KMAP for a variety of interesting habitats:

(ec_id:3.1.1.3. OR ec_id:3.1.1.101 OR ec_id:3.1.1.102 OR ec_id:3.1.1.74 OR ec_id:3.2.1.4 OR ec_id:3.2.1.1 OR ec_id:3.2.1.2 OR ec_id:3.2.1.15 OR ec_id:3.4.21.62 OR ec_id:4.2.2.2 OR ec_id:1.11.1.6) AND blast_pid:{60 TO *} AND blast_cov:{90 TO *} NOT ec_id:2.* NOT ec_id:5.*

PETase query for a search in KMAP Compare Module

ec_id:3.1.1.101 AND hmm_id:PF01738 AND blast_pid:{60 TO *} AND blast_cov:{80 TO *}

Antibiotic Resistance Genes query for KMAP Compare Module

1. (ko_id:K18215 OR ko_id:K18220 OR ko_id:K18221 OR ko_id:K18698) AND blast_pid:{60 TO *} AND blast_cov:{80 TO *}
2. (filter:F.AntiBiotic.Resistance) AND blast_pid:{60 TO *} AND blast_cov:{80 TO *}

KMAP TSV format conversion to biome

$ wget https://www.cbrc.kaust.edu.sa/aamg/KMAP_Data/KMAPtsv2Biome.Example.tgz

$ tar -xzvf KMAPtsv2Biome.Example.tgz

$ cd KMAPtsv2Biome.Example/

$ module purge && module load miniconda && pip install Cython --user && pip install argh --user && pip install biom-format --user && python kmap_tsv2biom.py tobiom --help

$ curl "https://www.cbrc.kaust.edu.sa/aamg/1525982760592_eBeachSand_50.0_intikhab/WWWAAMG/eBeachSand/metarep/eBeachSand.tsv.gz" -o eBeachSand.tsv.gz

$ gunzip eBeachSand.tsv.gz

$ python kmap_tsv2biom.py tobiom --annotfile eBeachSand.tsv --annotid koid -o eBeachSand_ko.biom
